# Application of an MTT-Based Colorimetric Assay to Evaluate SHED Metabolites in Inhibiting Oral Bacteria Growth

**DOI:** 10.1055/s-0045-1812497

**Published:** 2026-02-03

**Authors:** Oki Fadhila, Rini Devijanti Ridwan, Wahidah Tsamara Putri Yastuti, Yuliati, Indeswati Diyatri, Sellyn Angelina Margaretha, Huda Rashad Qaid, Mohammed Aljunaid, Thufailah, Chanaya Miranda Riveira

**Affiliations:** 1 Department of Oral Biology, Faculty of Dental Medicine, Universitas Airlangga, Surabaya, Indonesia; 2 Bachelor of Dental Science Program, Faculty of Dental Medicine, Universitas Airlangga, Surabaya, Indonesia; 3 Doctoral Program, Faculty of Dental Medicine, Universitas Airlangga, Surabaya, Indonesia; 4 Faculty of Oral and Dental Medicine, Department of Dentistry, Al-Saeed University, Taiz, Yemen; 5 Department of Oral and Dental Medicine, Faculty of Medicine, Taiz University, Taiz, Yemen

**Keywords:** medicine, inhibition, stem cells from human exfoliated deciduous metabolites, hydroxyapatite, oral bacteria

## Abstract

**Objective:**

Dental caries and periodontitis are among the most prevalent oral diseases, primarily caused by *Streptococcus mutans, Lactobacillus acidophilus, Aggregatibacter actinomycetemcomitans* , and *Porphyromonas gingivalis* . Metabolites from stem cells from human exfoliated deciduous teeth (SHED) expressing human β-defensin 4 exhibit antimicrobial effects. Chitosan-based hydrogels and hydroxyapatite (HA) have also shown antibacterial potential. This study aims to evaluate the antibacterial activity of hydrogels combined with SHED metabolites and HA against oral pathogens using amicroculture tetrazolium technique (MTT)-based colorimetric assay.

**Materials and Methods:**

Hydrogels were combined with SHED metabolites and/or HA, then applied to bacterial cultures. Viability was assessed via MTT assay and absorbance was measured using an enzyme-linked immunosorbent assay reader.

**Statistical Analysis:**

Data were analyzed using one-way analysis of variance (ANOVA) and Welch ANOVA ( *p* < 0.05).

**Results:**

The group treated with hydrogel + HA + SHED metabolites (K4) showed the lowest bacterial viability across all strains, with statistically significant differences ( *p* < 0.05).

**Conclusion:**

Hydrogels combined with SHED metabolites and HA demonstrate promising antibacterial properties and may serve as effective agents in preventing oral infections.

## Introduction

The etiology of caries is based on the four-factor theory: oral microorganisms, oral environment, host, and time. 1 The mechanism of the caries starts from endogenous bacteria, *Streptococcus mutans* and *Lactobacillus acidophilus* in the biofilm, producing acids from carbohydrate fermentation metabolism. This acid causes a low oral cavity pH and demineralization. 2 *Streptococcus mutans* is a group of facultative anaerobic gram-positive bacterium and is nonmotile. The cariogenic potential of *S. mutans* includes its ability to synthesize extracellular glucan polymers from sucrose, which helps the colonization of other bacteria in the development of the extracellular polymer matrix. *Lactobacillus acidophilus* is a facultative anaerobic gram-positive bacterium with rod-shaped cells that form a short chain. It is nonmotile and nonspore forming bacterium. These bacteria possess the capacity to convert carbohydrates into organic acids and can thrive at low pH levels. 3

Periodontitis is a chronic bacterial infectious disease that occurs in the tooth-supporting tissues including gingiva, periodontal ligament, and alveolar bone due to the interaction of pathogenic bacteria, composition of the subgingival biofilm, a damaged host immune response, and environmental factors. 4 In particular, severe damage from periodontal inflammation to the alveolar bone results in the loss of tooth support, which eventually causes the tooth to fall out. The main bacteria that cause periodontitis are *Aggregatibacter actinomycetemcomitans* and *Porphyromonas gingivalis* . 5 *Aggregatibacter actinomycetemcomitans* is a facultative anaerobic gram-negative bacterium that is shaped like a small rod, with a size 0.4 to 0.5 μm × 1.0 to 1.5 μm, nonmotile, and grow well at 5% CO _2._ 6 *Porphyromonas gingivalis* is an anaerobic gram-negative, rod-shaped, nonmotile bacterium that forms black colonies on blood agar media. This bacterium is detected high in areas where periodontitis occurs and detected low or even absent in healthy periodontal tissue. This bacterium plays a major role in the pathogenesis of periodontal disease, primarily inhabiting the subgingival sulcus of the oral cavity. 7

Stem cells have the ability to reproduce over a long period of time. In the bone marrow, there are two types of stem cells, namely, hematopoietic stem cells and bone marrow stromal stem cells (mesenchymal stem cells). Mesenchymal stem cells have the ability to repair and heal tissue because they have an immunomodulatory effect that can secrete angiopoietin-1, matrix metalloproteinase-3, and matrix metalloproteinase-9. There is also vascular endothelial growth factor and epidermal growth factor, which play a role in endothelial proliferation. 8 Mesenchymal stem cells have been shown to have therapeutic capabilities through their metabolites. Metabolites are substances involved in metabolism, which can be substances needed in metabolism or metabolic products. 9

One source of dental mesenchymal stem cells is stem cells from human exfoliated deciduous (SHED). SHED are heterogeneous cells isolated from the primary teeth of children aged 7 to 8 years. 10 SHED also has a metabolite that expresses human β-defensin 4 (HBD4) peptide in the defensin family and is known for its antimicrobial capabilities. 11 SHED shows outstanding potential for tissue remineralization. SHEDs are considered unique because they are taken from primary teeth that were previously thought to have no benefit. 12

SHEDs taken from the passage after subculture were used for cell viability testing against various bioinductive materials. Each passage involves separating and replanting the stem cells in a new culture vessel with fresh nutrient medium. This indicates that SHEDs had been cultured and passaged multiple times before being used in the experiments. 13 14 Growth factors influence the activation of the *HBD4* gene in SHED cells, triggering the production and secretion of HBD4, which have functions in modulating inflammatory responses and cell differentiation. 11 In addition, SHED also secretes metabolites that have been shown to have a therapeutic effect. 12 15

Hydroxyapatite (HA) is the main inorganic component of many hard tissues (HAP, Ca _10_ (PO _4_ ) _6_ (OH) _2_ ). 16 Hydrogel is a gel consisting of an aqueous dispersion phase with an appropriate hydrophilic gelling agent content. Hydrogels can be made from chitosan and beta-glycerol. Chitosan has antibacterial properties that can inhibit the growth of pathogenic bacteria including gram-positive and gram-negative bacteria. Glycerol forms hydrogen bonds between and within the chitosan chains, and “caps” the polymer chains, thereby protecting the chitosan from degradation, especially that caused by heat sterilization, increasing the solubility of chitosan at physiological pH, and facilitating the transition from solution to gel according to temperature. 17 The microculture tetrazolium technique (MTT)-based colorimetric assay is considered the gold standard to see the biocompatibility of materials and to determine the bioavailability of bacterial method is expected to be an effective choice for inhibiting the growth of oral bacteria.

## Materials and Methods

### Ethical Approval

The approval of this study was obtained by the Ethics Commission of the Faculty of Dental Medicine, Universitas Airlangga, Surabaya, Indonesia (reference number 113/HRECC.FODM/X/2023 and 1173/ HRECC.FODM/X/2023).

### Preparation of SHED Metabolites

SHED metabolites were purified from the naturally exfoliated human deciduous teeth provided by the Research Centre, Faculty of Dental Medicine, Universitas Airlangga. The SHED was cultured from passages 3 in Dulbecco's modified eagle medium. SHED culture medium was purified using the dialysis method to remove waste products of metabolism that were not useful, resulting in beneficial results of metabolites that contained several cytokines, growth factors, and exosomes.

### Preparation of Hydrogel

The 2.0% (w/v) chitosan solution was made by dissolving a certain amount of chitosan powder in 0.1 M acetic acid solvent. Then, a 56% (w/v) β-glycerophosphate solution is made by dissolving the β-glycerophosphate powder into deionized water to make the required volume and concentration, and then, the mixture is sterilized through a 0.22-μm filter. After that, the β-glycerophosphate solution was added slowly to the chitosan solution that had been prepared previously in an ice bath with a CH:GP volume ratio of 5:1, and after stirring, the CH:GP mixture was stored at 37°C.

### Preparation Hydroxyapatite Paste

HA paste is made by adding HA powder to deionized water and ultrasonicating it until it becomes a dispersed HA paste. HA paste is made with a 15% concentration.

### Preparation of Hydrogel Combination with SHED Metabolites

SHED metabolites were dropped slowly into the CH:GP mixture. 18 The ratio used for CH:GP: SHED metabolites are 5:1:1. The treatment was carried out in an ice bath with a stirrer at 500 rpm until homogeneous, then put it in a syringe and stored it in the freezer at 4°C.

### Preparation of Hydrogel Combination with Hydroxyapatite

The dispersed HA paste was added to the CH:GP mixture (with a CH:GP:HA ratio 5:1:1) in a water bath with a CH:GP:HA volume ratio of 5:1:1, 18 then it was put into a syringe and stored in a freezer at 4°C.

### Preparation of Hydrogel Combination with Hydroxyapatite and SHED Metabolites

The primary tooth stem cell metabolite slowly dripped into the CH:GP:HA mixture. 18 The ratio used was CH:GP:HA: SHED metabolites is (5:1:1:1). The treatment was carried out in an ice bath with a stirrer at 500 rpm until homogeneous. Then, put it into a syringe and store in a freezer at 4°C.

### Bacterial Culture and MTT-Based Colorimetric Assay

*Streptococcus mutans* (ATCC 25175), *L. acidophilus* (ATCC 4356), *A. actinomycetemcomitans* (ATCC 43718), and *P. gingivalis* (ATCC 33277) is cultured in brain heart infusion-broth (BHI-B) media, and then incubated at 37°C for 2 × 24 hours. The bacterial suspension was adjusted to the McFarland 0.5 standard and then put into different well plates with sample K1 (hydrogel), sample K2 (hydrogel combined with SHED metabolite), sample K3 (hydrogel combined with HA), sample K4 (hydrogel combined with HA and SHED metabolite), bacterial control (KB) is a control group containing bacteria without any treatment, and media control (KM) contains medium treatment without bacteria. Each of the six samples was incubated for 24 hours at a temperature of 37°C. After that, 10 µl MTT solution was added to each plate and incubated again for 4 hours at 37°C. Next, take a sample from each plate that is given 100 µl of NaOH solution. The absorbance of the formazan product was calculated by measuring it at a wavelength of 550 nm using an enzyme-linked immunosorbent assay (ELISA) reader. The absorbance value is converted into a percentage using the formula:



### Statistical Analysis

SPSS (IBM, Chicago, Illinois, United States) version 21 was used for statistical analysis. Viability of the bacteria data is shown in the form of mean ± standard deviation. One-way analysis of variance (ANOVA) and one-way ANOVA Welch were conducted to find differences between groups ( *p* < 0.05).

## Results

**Fig. 1:**
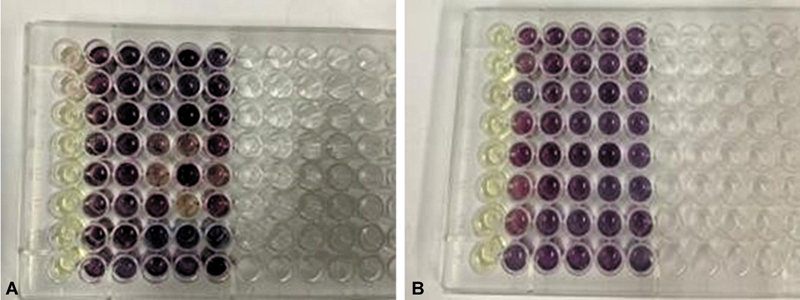
Bacterial samples on 96-well plate. ( **A** ) *S. mutans* and ( **B** ) *L. acidophilus* .

*Streptococcus mutans* and *L. acidophilus* groups were used 96-well plates, with 16-well plates used for media control, 16-well plates used for cell control, and other 64-well plates for the treatment group ( Fig. 1 ). Samples were then examined using the MTT method, and the absorbance value was measured by an ELISA reader at a wavelength of 550 nm.

**Fig. 2:**
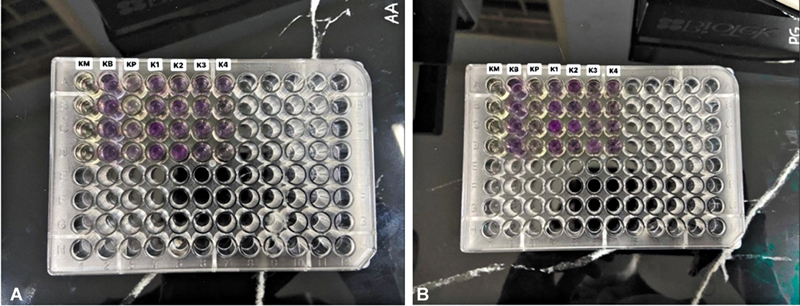
Bacterial samples on 56-well plate. ( **A** ) *A. actinomycetemcomitans* and ( **B** ) *P. gingivalis* .

*Aggregatibacter actinomycetemcomitans* and *P. gingivalis* groups were used in 56-well plates, with 8-well plates used for media control, 8-well plates used for cell control, and other 40-well plates for treatment group ( Fig. 2 ). Samples were then examined using the MTT method, and the absorbance value was measured by an ELISA reader at a wavelength of 550 nm.

Based on Table 1 , the lowest average absorbance value of the *S. mutans* absorption from the treatment group is the K4 group (1.30 ± 0.18), which has a viability of 65.97%. Meanwhile, in *L. acidophilus* , the lowest absorbance is 1.40 ± 0.07 in the K4 group with 70.94% viability. This means that hydrogel combined with HA and SHED metabolite has an inhibition effect on the growth of *S. mutans* by 34.02% and *L. acidophilus* by 29.05%. The results of the one-way ANOVA test showed a significance value of 0.000, meaning that *p* -value <0.05. This shows that there is an inhibitory effect of treatment on the growth of *S. mutans* and *L. acidophilus.*

**Table 1:** Absorbance values and viability of *S. mutans* and *L. acidophilu* *s*

Groups	*S. mutans* absorbance value Mean ± SD	*S. mutans* viability (%) Mean	*p* -Value	*L. acidophilus* absorbance value Mean ± SD	*L. acidophilus* viability (%) Mean	*p* -Value
KM	0.11 ± 0.04	0	0.000	0.06 ± 0.019	0	0.000
KB	1.91 ± 0.35	100		1.94 ± 0.25	100	
K1	1.76 ± 0.21	91.79		1.56 ± 0.44	79.41	
K2	1.43 ± 0.33	73.41		1.44 ± 0.05	73.19	
K3	1.49 ± 0.13	76.84		1.49 ± 0.15	76.08	
K4	1.30 ± 0.18	65.97		1.40 ± 0.07	70.94	

Abbreviations: SD, standard deviation; SHED, stem cells of human exfoliated deciduous.

Notes: K1—hydrogel group. K2—hydrogel combined with SHED metabolite group. K3—hydrogel combined with hydroxyapatite group. K4—hydrogel combined with hydroxyapatite and SHED metabolite group. KB—bacterial control group, containing bacteria without any treatment. KM—media control group, contains medium treatment without bacteria.

MTT testing ( Table 2 ) against *A. actinomycetemcomitans* showed that the K4 group has the lowest absorbance value (206 ± 7.21) with viability 67.96%. Meanwhile, *P. gingivalis* has the lowest absorbance value 135.67 ± 6.51 in K4 group and viability 44.78%. Based on the results, hydrogel combined with HA and SHED metabolite has an inhibition ability against *A. actinomycetemcomitans* by 32.04% and *P. gingivalis* by 55.22%. The results of the ANOVA Welch's test showed a significant value of 0.003, meaning that *p* -value <0.05. This shows that there is an inhibitory effect of treatment on the growth of *A. actinomycetemcomitans* and *P. gingivalis.*

**Table 2:** Absorbance values and viability of *A. actinomycetemcomitans* and *P. gingivalis*

Groups	*A. actinomycetem* comitans absorbance value Mean ± SD	*A. actinomycetem* comitans viability (%) Mean	*p* -Value	*P. gingivalis* absorbance value Mean ± SD	*P. gingivalis* viability (%) Mean	*p* -Value
KM	0.11 ± 0.04	0	0.000	0.06 ± 0.019	0	0.000
KB	1.91 ± 0.35	100		1.94 ± 0.25	100	
K1	1.76 ± 0.21	91.79		1.56 ± 0.44	79.41	
K2	1.43 ± 0.33	73.41		1.44 ± 0.05	73.19	
K3	1.49 ± 0.13	76.84		1.49 ± 0.15	76.08	
K4	1.30 ± 0.18	65.97		1.40 ± 0.07	70.94	

Abbreviations: SD, standard deviation; SHED, stem cells of human exfoliated deciduous.

Notes: K1—hydrogel group. K2—hydrogel combined with SHED metabolite group. K3—hydrogel combined with hydroxyapatite group. K4—hydrogel combined with hydroxyapatite and SHED metabolite group. KB—bacterial control group, containing bacteria without any treatment. KM—media control group, contains medium treatment without bacteria.

## Discussion

Inhibition activity is one of the important factors that must be studied to determine the ability of a material to inhibit bacterial growth. This study used SHED metabolite hydrogel with HA to analyze the inhibitory effect of the material on the growth of oral bacteria. In the test, the MTT-based colorimetric assay was used for assessing the bacterial viability of *S. mutans* , *L. acidophilus* , *A actinomycetemcomitans* , and *P. gingivalis* . The MTT absorption value will increase along with the number of living bacterial cells. The decrease in bacterial cell viability indicates the SHED metabolite hydrogel material with HA has the ability to inhibit the growth of *S. mutans* , *L. acidophilus* , *A. actinomycetemcomitans* , and *P. gingivalis* . In this study, it was found that all the groups had the ability to inhibit these bacteria with varying percentages. The results for the four bacteria tested showed that the viability was the lowest in the K4 group, which consisted of hydrogel combined with HA and SHED metabolite.

The ability of hydrogel to inhibit bacterial growth has been proven by the research, which explains that the active ingredients of hydrogel, namely chitosan, were able to inhibit gram-positive bacteria and explain how chitosan inhibits gram-positive bacteria. 19 20 The mechanisms of chitosan in inhibiting gram-positive bacteria by interacting with lipids in the bacterial cell wall and enabling chitosan bind to microbial DNA. Then, it disrupts mRNA and protein synthesis and finally causes bacterial lysis. The previous research proves the ability of SHED metabolite to inhibit bacterial growth by expressing HBD4, which has a direct effect against some bacteria as well as antimicrobial peptides. 9 The positively charged HBD4 will interact with the gram-positive bacterial cell membrane, which is negatively charged. 21 The peptide-lipid ratio in the bacterial cell membrane will increase, allowing HBD4 to penetrate the membrane and enter the cytoplasm of the bacterial cell. HBD4 then interacts with intercellular components and inhibits the synthesis of DNA, RNA, proteins, and enzyme activity. HBD4 also induces the release of lyases, which have the ability to break down the bacterial cell structure.

HA has an antimicrobial effect by bonding with the pellicle on the tooth surface and inhibiting adhesins on bacterial cell walls. When bacterial adhesin is blocked by HA, the bacteria have difficulty attaching to the tooth pellicle. The inhibition of gram bacteria growth by HA has been positively proven by the research 22 and by showing that while Ca ^2+^ ions from HA interact with the cell membrane and increasing the membrane permeability resulting in osmotic shock. Air enters the cell because of osmotic shock, which leads the cell to swell and lyse. This condition will cause damage and death of bacterial genetic material. The differences in the ability of materials to inhibit the growth of all the oral bacteria compared to each other are possibly caused by the characteristics of each bacterium itself. 23

These findings support the potential clinical translation of this biomaterial as an innovative prevention and treatment of dental materials by inhibiting oral bacteria growth, particularly in preventive dentistry, as a coating or additive in restorative materials (e.g., fillings, liners) to inhibit bacterial colonization and recurrent caries. Additionally, periodontal therapy, as a local drug delivery system or scaffold in guided tissue regeneration procedures to reduce periodontal pathogens and promote healing. Postsurgical wound care, to prevent infections after dental extractions, implants, or periodontal surgeries.

This study only tested a few types of bacteria ( *S. mutans* , *L. acidophilus* , *A. actinomycetemcomitans* , and *P. gingivalis* ). However, in clinical conditions, many other bacterial species also play a role in the pathogenesis of caries and periodontitis. Therefore, the results cannot be generalized to all oral bacteria. This study was conducted under laboratory conditions ( *in vitro* ), which do not fully represent the complexity of the human oral cavity environment, such as microbial interactions, salivary flow, dynamic pH, and the local immune system. The effectiveness of the test materials under *in vivo* conditions may vary.

## Conclusion

Based on MTT assay results, hydrogels combined with SHED metabolites and HA effectively inhibited the growth of major oral pathogens, including *S. mutans* , *L. acidophilus* , *A. actinomycetemcomitans* , and *P. gingivalis* . These findings highlight their potential for therapeutic application in oral health.
